# Correction: Pharmacist-led educational interventions provided to healthcare providers to reduce medication errors: A systematic review and meta-analysis

**DOI:** 10.1371/journal.pone.0294195

**Published:** 2023-11-03

**Authors:** Myriam Jaam, Lina Mohammad Naseralallah, Tarteel Ali Hussain, Shane Ashley Pawluk

There are errors in the Funding section. The correct Funding statement is: Open Access funding provided by the Qatar National Library.

## References

[1] Jaam M, Naseralallah LM, Hussain TA, Pawluk SA (2021) Pharmacist-led educational interventions provided to healthcare providers to reduce medication errors: A systematic review and meta-analysis. PLoS ONE 16(6): e0253588. doi: 10.1371/journal.pone.0253588 34161388 PMC8221459

