# Supplementary material for: Pharmacist-led educational interventions provided to healthcare providers to reduce medication errors: A systematic review and meta-analysis
Source: PLoS One. 2021 Jun 23;16(6):e0253588. doi: 10.1371/journal.pone.0253588 (PMC8221459; doi:10.1371/journal.pone.0253588)
Supplement: S1 File — (DOCX) [file pone.0253588.s002.docx]

**S1 File**

**Search strategy**

| Cochrane | Pharma* AND (Medication errors OR Adverse effect OR inappropriate prescribing OR safe prescribing OR medication discrepancy OR medication safety OR adverse drug event) AND (Prevent* OR Reduc* OR decrease OR augment OR effectiveness OR improvement) in Abstract in Cochrane Reviews' | Abstract | 2889 |
| --- | --- | --- | --- |
|  |  |  |  |
| EMBASE | **'pharma*'**:ab,ti AND (**'medication errors'**:ab,ti OR **'adverse effect'**:ab,ti OR **'mistake'**:ab,ti OR **'inappropriate prescribing'**:ab,ti OR **'safe prescribing'**:ab,ti OR **'medication discrepancy'**:ab,ti OR **'medication safety'**:ab,ti OR **'adverse drug event'**:ab,ti) AND (**'prevent*'**:ab,ti OR **'reduc*'**:ab,ti OR **'decrease'**:ab,ti OR **'augment'**:ab,ti OR **'effectiveness'**:ab,ti OR **'improvement'**:ab,ti) | Abstract, title | 2831 |
|  |  |  |  |
| EBSCO | Pharma* AND (Medication errors OR Adverse effect OR inappropriate prescribing OR safe prescribing OR medication discrepancy OR medication safety OR adverse drug event) AND (Prevent* OR Reduc* OR decrease OR augment OR effectiveness OR improvement) in Abstract in Cochrane Reviews' | English, articles, published since 2000 | 2691 |
| Medline | ((Pharma*[Title/Abstract]) OR "Pharmacy"[Mesh]) AND ((Medication errors[Title/Abstract] OR ("Medication Errors"[Mesh][Title/Abstract]) OR Adverse effect[Title/Abstract] OR ("drug-related side effects and adverse reactions"[Mesh][Title/Abstract]) OR ("Drug-Related Side Effects and Adverse Reactions"[Mesh][Title/Abstract]) OR inappropriate prescribing[Title/Abstract] OR safe prescribing[Title/Abstract] OR medication discrepancy[Title/Abstract] OR medication safety[Title/Abstract] OR adverse drug event)[Title/Abstract])) AND ((Prevent*[Title/Abstract] OR Reduc*[Title/Abstract] OR decrease[Title/Abstract] OR augment[Title/Abstract] OR effectiveness[Title/Abstract] OR improvement[Title/Abstract])) AND (Education[Title/Abstract] OR training[Title/Abstract] OR teach[Title/Abstract]) | English, articles, published since 2000 | 935 |


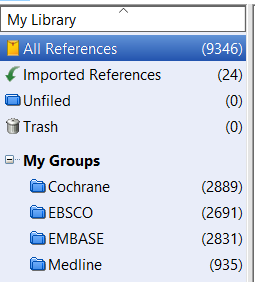


**Full Screening files are available through this link:**

[**https://docs.google.com/spreadsheets/d/1AzJOasm_1VAlK-RmhRZa4wvS0D2Y9-21QTvtTKnbfBs/edit?usp=sharing**](https://docs.google.com/spreadsheets/d/1AzJOasm_1VAlK-RmhRZa4wvS0D2Y9-21QTvtTKnbfBs/edit?usp=sharing)
