# Supplementary material for: Pharmacist-led educational interventions provided to healthcare providers to reduce medication errors: A systematic review and meta-analysis
Source: PLoS One. 2021 Jun 23;16(6):e0253588. doi: 10.1371/journal.pone.0253588 (PMC8221459; doi:10.1371/journal.pone.0253588)
Supplement: Attachment — Submitted filename: Response to Reviewers.docx [file pone.0253588.s003.docx]

May 2, 2021

The Editor-in-Chief

PLOS ONE

Dear Madam/Sir,

**Revision of Manuscript PONE-D-21-05743: Pharmacist-led educational interventions provided to healthcare providers to reduce medication errors: A systematic review and meta-analysis**

The authors of the above-named manuscript would like to thank you for your e-mail dated 27 April 2021, containing the constructive comments of the reviewer. The authors very much appreciate the comments and have revised the manuscript accordingly as attached. We are positive that the comments helped in enhancing the quality and scientific merit of the paper.

Below are separate pages, detailing our responses to the comments of the reviewer.

**Please note that changes are made using “track changes” tool within the revised manuscript.**

Thank you to the editor and the reviewer for the time spent on this manuscript. We look forward to receiving your kind response.

Sincerely yours,

**The Corresponding Author**

**All in all, we thank you for your great review which we believe would add great value to the adjusted manuscript.**

**Detailed Responses to the Comments of the Reviewer**

| Reviewer #1: REVIEWER’S COMMENTS | Author’s response |
| --- | --- |
| The topic is highly relevant. To a large extent, the report followed the Preferred Reporting Items for Systematic revies and Meta-Analyses (PRISMA) Checklist. However, the manuscript needs significant editing for language and writing quality, to eliminate ambiguities. This would improve the fluidity and readability of the text. The authors should provide more explanations to make the Discussion section clearer. Readers might appreciate this section better, if comparisons/contrasts are made between their findings and those from similar studies while bringing to the fore the relevance in pharmacy practice. The manuscript could be accepted for publication if the necessary corrections are made in the revised submission. | The authors thank the reviewer for their valuable comment. The article was reviewed for language and writing with adjustments made throughout. Additionally, the discussion was adjusted to accommodate the reviewer’s comment which we believe adds value to the manuscript. |
| ABSTRACT 1. Line 53: Write the full meaning of PROSPERO with the abbreviation in bracket. End the sentence with a full stop. 2. Line 54: … twelve (12) studies … (It is usually better not to start a sentence with figures) 3. Line 65: Preferably, arrange the keywords in alphabetical order separated by semi-colons. 4. General: Abstract is clear and gives a good description of the study. | All comments were addressed |
| INTRODUCTION 1. Line 70: Medication errors are defined … 2.Line 81: “safety” was mentioned twice 3. Lines 85 & 95: You can rephrase the statements without including the authors’ names. 4. Line 90: … have been reported to increase … 5 Line 91: … at the same time, reducing the … 6. Line 98: … aimed to … | All comments were addressed |
| MATERIALS AND METHODS 1. Line 103: The authors need to write the full meaning of PRISMA with the abbreviation in brackets. The authors should cite one of the original publications of the PRISMA Statement or PRISMA Explanation and Elaboration. 2. Line 104: Write the full meaning of PROSPERO with the abbreviation in brackets (In addition, correct the spelling of your abbreviation). The authors need to provide citation or where the review protocol can be accessed. 3. Line 112: … two researchers, independently, to ensure … 4. Line 114: … two authors, independently OR two independent authors. 5. Line 115: Category C – Did the authors mean to write “Reduce*”? 6. Lines 118 & 119: For clarity of the inclusion criteria, it needs to be rephrased. The authors can opt for the use of lower-case Roman numerals. 7. Line 120: It is unnecessary to include “regardless of study design” as this creates ambiguity. 8. Lines 121 – 124: For clarity of the exclusion criteria, the authors can use lower-case Roman numerals for the listing. 9. Line 123: … led by students … 10 Line 131: … two independent researchers … 11. Line 133: Include citation for the Crowe Critical Appraisal Tool (CCAT) 12. Line 135: Each domain has a maximum score of five … (Write single digits as words) 13. Line 137: … two independent researchers … 14. Line 137/8: The CCAT was previously … | All comments were fully addressed. We also wish clarify in response to this comment: 5. Line 115: Category C – Did the authors mean to write “Reduce*”?  The authors used * to capture the following: Reduction, Reducing, Reduce. This was not a typo. |
| RESULTS 1. Line 149: … (n = 6857), the remaining articles … 2. Line 150: … two researchers. A total of 12 articles … 3. Line 166: Additionally, … 4. Line 167: Delete “or” 5. Table 2: The contents for the “medication error rate post-intervention” column is confusing. What do the figures after the duration in round brackets represent? Is this captured in the heading? 6. General: Be uniform in your report of the percentages and figures. Use either one decimal place or two decimal places. Be consistent with how you write post or pre-intervention. Is it post-intervention or postintervention? Be consistent. “et al.” should be italicized. 7. Line 176: CCAT appraised … 8. Line 183/4: … that varied in content … 9. Line 188: … two weeks … 10. Line 191: … six studies … 11. Line 204: … that summarized … 12. Line 214: The last statement needs to be rephrased. Starting a statement with “Nor” could confuse readers. 13. Line 218: Do you mean the ten studies presented that P-value of 0.0004? Was this stated in the studies or calculated by the authors? If any of the P-values were by the authors, then it should reflect in the Methods section (data analysis and synthesis). The level of significance should be included. | All comments were addressed, and the p value statistics was added in methods under data analysis |
| DISCUSSION 1. General: The Discussion section needs to be improved. The authors seemed to focus on making comparisons between the studies that were included in their study. This was similar to Results summary. A more robust Discussion should provide possible reasons for the findings; compare/contrast the findings with other studies; relate the findings to pharmacy practice. 2. Line 232 – 234: This should be in the Introduction section, as it is a justification for conducting the study. It should not appear in the Discussion section. The introductory paragraph of the Discussion should be a summary of the findings without repeating the results. 3. Line 237/8: Do not report figures in your Discussion section, as they should already be in the Results section. 4. Line 245: one month or 1-month 5. Line 267 – 269: Sentence should be rephrased. 6. Line 273 – 274: Sentence should be rephrased. It appears incomplete. 7. General: The use of different referencing styles should be corrected all through the manuscript. 8. Line 281 – 285: Sentence is complex and can be broken into simple sentences. For clarity, it is better to have two simple sentences than have one extremely long sentence with multiple punctuation marks and conjunctions. 9. Line 287: … In lieu, … 10. Line 289: … site-specific, based … 11. Line 291/2: The sentence should be rephrased. 12. Line 294: delete “All in all”. 13. Line 294: … the included studies … 14. Line 295: … CCAT … 15. Line 301: Write the full meaning of EQUATOR with the abbreviation in bracket. 16. Line 309: … was reported. (Delete “and was expected”) 17: General: The limitations were well described. 18. Line 340: Do you mean “To err is human”? | The authors appreciate the comments provided by the reviewer. All comments were addressed in the revised manuscript. |
| CONCLUSION 1. Line 348/9: It is not necessary to include the overall odds ratio in the Conclusion. 2. Line 350: … that summarize  CONTRIBUTIONS Line 365 – 368: All authors should be mentioned. All authors should be represented by their full abbreviations.  REFERENCES 1. Please follow the authors’ guidelines. There should be uniformity in the References. For instance, some Journal titles are abbreviated while others are not e.g., Reference 4, 11, 42. Check all. 2. Reference 40, 46: Include date cited. | All comments were fully addressed in the revised manuscript. |
| FIGURES 1. Figure 1: The descriptions on the far-left are upside down. 2. Figure 1: The calculation is confusing as (9346 + 8) – 6857 = 2497 (The authors’ flow diagram shows 2498). The first sentence in the Results section confirms that 9354 articles were identified from the different databases and reference screening. The correct values should be inputted in the flow diagram. | The comments were addressed in the figure. |

| Reviewer #2: REVIEWER’S COMMENTS | Author’s response |
| --- | --- |
| I have several suggestions for improving your manuscript. Abstract 1.Please write the sampling method, study design, and study time.  Introduction 2. I think it is better to remove the following sentence and add it to the end of the introduction before stating the purpose. " This study sought to implement a remote corporate-based pharmacist into a home-based primary care practice to facilitate comprehensive medication reviews." 3. Please provide the references in the following sentences. Line:79, 97, 4. At the end of line 175, change "a American…" to " an American….".  Methods 5. Please write the sampling method, study design, and study time. 6. Please explain inclusion and exclusion criteria. 7. Please write ethical considerations.  8. In Table 1, the total percentage in the payer's column is 99.9%, please correct. 9. In table 1, the term Std should be clarified. 10. Please use the full form of "VTE" first used in table 3. Discussion 11. You can suggest conducting experimental studies such as Randomised Clinical Trial (RCT) for future studies. Best regards | We thank the reviewer for their valuable comments. All comments provided were addressed. |
