# Supplementary material for: Pharmacist-led educational interventions provided to healthcare providers to reduce medication errors: A systematic review and meta-analysis
Source: PLoS One. 2021 Jun 23;16(6):e0253588. doi: 10.1371/journal.pone.0253588 (PMC8221459; doi:10.1371/journal.pone.0253588)
Supplement: Attachment — Submitted filename: Response to Reviewers.docx [file pone.0253588.s004.docx]

May 30, 2021

The Editor-in-Chief

PLOS ONE

Dear Madam/Sir,

**Revision of Manuscript PONE-D-21-05743R1: Pharmacist-led educational interventions provided to healthcare providers to reduce medication errors: A systematic review and meta-analysis**

The authors of the above-named manuscript would like to thank you for your e-mail dated 26 May 2021, containing the constructive comments of the reviewer. The authors very much appreciate the comments and have revised the manuscript accordingly as attached. We are positive that the comments helped in enhancing the quality and scientific merit of the paper.

Below are separate pages, detailing our responses to the comments of the reviewer.

**Please note that changes are made using “track changes” tool within the revised manuscript.**

Thank you to the editor and the reviewer for the time spent on this manuscript. We look forward to receiving your kind response.

Sincerely yours,

**The Corresponding Author**

**All in all, we thank you for your great review which we believe would add great value to the adjusted manuscript.**

**Detailed Responses to the Comments of the Reviewer**

| Journal Requirements: | Author’s response |
| --- | --- |
| Please review your reference list to ensure that it is complete and correct. If you have cited papers that have been retracted, please include the rationale for doing so in the manuscript text, or remove these references and replace them with relevant current references. Any changes to the reference list should be mentioned in the rebuttal letter that accompanies your revised manuscript. If you need to cite a retracted article, indicate the article’s retracted status in the References list and also include a citation and full reference for the retraction notice. | The authors have reviewed each reference and added the DOI whenever applicable. |
| Reviewer #1: |  |
| GENERAL COMMENTS  The revised manuscript addressed the concerns in the first revision. The manuscript should be accepted for publication after the few corrections, stated below, are effected.  MATERIALS AND METHODS  Lines 103: meta-analyses (more appropriate in its plural form)  DISCUSSION  1. Line 228: et al.,  2. Line 303: Why is this line empty?  CONTRIBUTIONS  Line 346: Contributions  REFERENCES  Include date cited for accessed websites in References 40 & 46. | The authors thank the reviewer for their comments. The authors addressed all comments mentioned. |
| Reviewer #2: |  |
| Thank you for your high-quality work. I have some minor comments for improving your manuscript.  1. In line 60 you stated " Studies reporting...." and mentioned only one reference, I checked the reference. Considering that the article is a systematic review study, it is better instead of " Studies reporting... " write" the results of a systematic review .... ". Because we are expected to refer to a number of articles when we mention the word "studies".  2.The introduction is well organized and follows a logical course. However, I suggest that it is better to mention the statistics of medication errors in the introduction section (about two or three sentences).  3.Please check the numbers in Table 3 again. The Overall Out of 40 of Fawaz et al study [21] is 23.5%, which is calculated in the table 25.5%.  4.Please correct the following:  Line 22: "increased" to " an increased or the increased " be corrected.  In line 143, the means of term "hospital" in the sentence "three covered emergency/acute care hospital" Is it hospital wards or hospitals?  Best regards | The authors appreciate the comments provided. All comments were addressed in the revised manuscript. More statistics relevant to the topic were added in the introduction.  All numbers were reviewed within table 3 to ensure accuracy. Modification was made to correct the error.  All other comments were fully addressed. |
